# The cerebellum for jocks and nerds alike

**DOI:** 10.3389/fnsys.2014.00113

**Published:** 2014-06-17

**Authors:** Laurentiu S. Popa, Angela L. Hewitt, Timothy J. Ebner

**Affiliations:** Department of Neuroscience, University of MinnesotaMinneapolis, MN, USA

**Keywords:** performance errors, sensory prediction errors, internal models, Purkinje cells, cognition

## Abstract

Historically the cerebellum has been implicated in the control of movement. However, the cerebellum's role in non-motor functions, including cognitive and emotional processes, has also received increasing attention. Starting from the premise that the uniform architecture of the cerebellum underlies a common mode of information processing, this review examines recent electrophysiological findings on the motor signals encoded in the cerebellar cortex and then relates these signals to observations in the non-motor domain. Simple spike firing of individual Purkinje cells encodes performance errors, both predicting upcoming errors as well as providing feedback about those errors. Further, this dual temporal encoding of prediction and feedback involves a change in the sign of the simple spike modulation. Therefore, Purkinje cell simple spike firing both predicts and responds to feedback about a specific parameter, consistent with computing sensory prediction errors in which the predictions about the consequences of a motor command are compared with the feedback resulting from the motor command execution. These new findings are in contrast with the historical view that complex spikes encode errors. Evaluation of the kinematic coding in the simple spike discharge shows the same dual temporal encoding, suggesting this is a common mode of signal processing in the cerebellar cortex. Decoding analyses show the considerable accuracy of the predictions provided by Purkinje cells across a range of times. Further, individual Purkinje cells encode linearly and independently a multitude of signals, both kinematic and performance errors. Therefore, the cerebellar cortex's capacity to make associations across different sensory, motor and non-motor signals is large. The results from studying how Purkinje cells encode movement signals suggest that the cerebellar cortex circuitry can support associative learning, sequencing, working memory, and forward internal models in non-motor domains.

## Introduction

The role of the cerebellum in the nervous system remains controversial. Traditionally, cerebellar function has been viewed in the context of motor control, given the well-established fact that cerebellar insults result in movement deficits. Different aspects of the cerebellum's involvement in motor control have generated multiple hypotheses regarding cerebellar function including movement timing (Braitenberg and Atwood, 1958; Keele and Ivry, 1990; Welsh et al., 1995; O'Reilly et al., 2008), error detection and correction (Oscarsson, 1980), motor learning (Marr, 1969; Albus, 1971; Gilbert and Thach, 1977; Ito, 2002), and providing internal models (Wolpert et al., 1998; Kawato, 1999; Imamizu et al., 2000; Morton and Bastian, 2006; Shadmehr et al., 2010).

More recently, evidence for cerebellar involvement in non-motor processes such as cognition, emotions and social interaction has accumulated at a rapid pace. The findings include the rapid expansion of the cerebellar hemispheres in primates (Leiner et al., 1986, 1989) with development of projections between the cerebellum and non-motor cortical areas (Schmahmann and Pandya, 1989, 1991, 1997; Middleton and Strick, 1994, 2001; Kelly and Strick, 2003), cerebellar activation related to cognitive behaviors (Petersen et al., 1988; Kim et al., 1994; Hayter et al., 2007), cognitive and emotional dysfunction associated with cerebellar lesions/disease (Fiez et al., 1992; Schmahmann, 2004; Burk, 2007), and the influence on cognitive processes by manipulating cerebellar excitability (Ferrucci et al., 2008; Pope and Miall, 2012; Boehringer et al., 2013). These contributions to non-motor behaviors raise the question of whether the cerebellum performs specific computations/functions in different domains or performs a common process across all domains. Based on its stereotypical architecture, a plausible and parsimonious hypothesis is that the cerebellum performs a uniform process in both motor and non-motor processes (Schmahmann, 2000, 2010; Ramnani, 2006; Thach, 2007; Ito, 2008). An important implication of this hypothesis is that understanding cerebellar information processing in the motor domain can illuminate the contributions of the cerebellum in non-motor domains.

Working from the common processing viewpoint, this review argues that the present state of our understanding of the cerebellum's role in motor behavior can shed light on non-motor functions. Several authors have taken a similar perspective (Ito, 2008; Imamizu and Kawato, 2009; Pezzulo, 2011; Pezzulo et al., 2012). An advantage of applying a motor control view to non-motor processing is that subjects, including non-human primates, can be required to perform extremely demanding motoric tasks in highly controlled environments. Despite task complexity, motor behavior can be described and quantified by well-defined measures, allowing intimate and unambiguous access to cerebellar signals. Therefore, this review evaluates the motor signals encoded by cerebellar neurons, including the existence of a new class of signals in Purkinje cell simple spike activity related to performance errors, as well as a dual encoding mechanism for motor parameters (Hewitt et al., 2011; Popa et al., 2012). Together, these signals could provide the neural substrate for computing sensory prediction errors postulated by internal model hypotheses (Wolpert and Ghahramani, 2000; Mazzoni and Krakauer, 2006; Shadmehr et al., 2010). We suggest these new findings regarding simple spike signaling in motor behaviors provide insights into cerebellar function that could help understanding cerebellar involvement in non-motor domains.

## Cerebellum and motor domain

### Kinematic signals

Numerous studies have documented that simple spike activity encodes kinematic variables, including position, velocity, speed and acceleration that describe body part motions without consideration of their causes such as forces or joint torques. This common encoding of kinematics is true for different effectors and motor behaviors. In the intermediate zone and its neighboring lateral zones surrounding the primary fissure, Purkinje cells encode position, direction, amplitude, velocity and speed of arm movements (Thach, 1970; Harvey et al., 1977; Mano and Yamamoto, 1980; Marple-Horvat and Stein, 1987; Fortier et al., 1989; Fu et al., 1997; Coltz et al., 1999; Roitman et al., 2005; Pasalar et al., 2006). These regions in the monkey are analogous to the regions in the human cerebellum engaged during limb movements (Stoodley and Schmahmann, 2009; Timmann et al., 2009). In the floccular complex, Purkinje cells encode eye position, velocity and acceleration during smooth pursuit and ocular following (Stone and Lisberger, 1990; Shidara et al., 1993; Gomi et al., 1998; Medina and Lisberger, 2009). In the posterior vermis, similar kinematic encoding of the eye movements is present for saccades and smooth pursuit (Thier et al., 2002; Dash et al., 2013). An important issue in interpreting these results is the possible confound between effector kinematics and kinetics (Shidara et al., 1993; Ebner et al., 2011). However, a study designed to eliminate this confound demonstrated that Purkinje cells do not encode kinetics or muscle activity during manual tracking (Pasalar et al., 2006). We conclude that the representations of kinematics in the simple spike activity are unambiguous and ubiquitous, suggesting that the cerebellar cortex performs common processing for very different movements and effectors.

However, other confounds present in some experimental paradigms have introduced ambiguity in the results. For example, saccadic eye movements, circular tracking, and center-out reach all introduce high degrees of correlation among kinematic parameters (Paninski et al., 2004; Hewitt et al., 2011). Further, most paradigms do not provide a uniform or complete coverage of the work space of kinematic variables. Another confound in assessing if cell firing leads kinematics, required of forward internal hypotheses (Miall and Wolpert, 1996; Bastian, 2006; Shadmehr et al., 2010), is the predictability found in many tasks. Task predictability introduces the possibility that leading simple spike activity could be due to expectations related to upcoming trials rather than predicting the consequences of the motor command.

**Figure 1:**
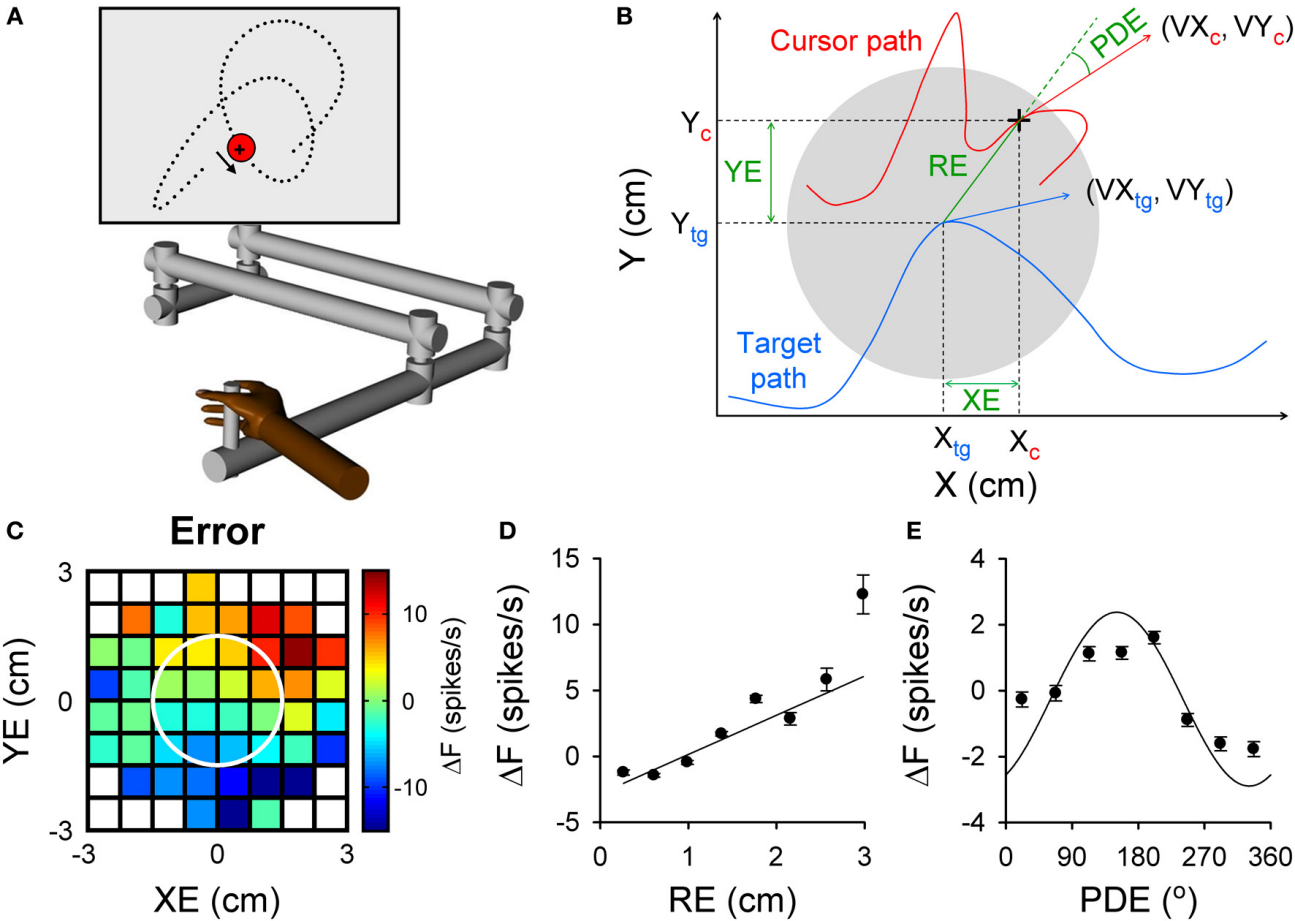
**Purkinje cell simple spike modulation in relation to task errors and hand kinematics during random tracking. (A)** Using a robot manipulandum, moving in an horizontal plane, monkeys track a randomly moving target on a vertical screen, by keeping the cross-shaped cursor within the target (3 cm in diameter). Excursions of the cursor outside target area longer than 500 ms result in trial abort. **(B)** Geometry of the motor parameters. Target (gray circle) moves on a random trajectory (blue trace). Cursor (black cross) maps the hand movement (red trace). Hand kinematic parameters are position (X_c_ and Y_c_), velocity (VX_c_ and VY_c_) and speed (magnitude of the hand velocity vector). Performance error parameters are position error (XE and YE) and RE (magnitude of the position error vector) and position director error (PDE—the difference between the direction of the position error vector and direction of hand movement). **(C–E)** Simple spike modulation for an example Purkinje cell is plotted in relation to performance errors: XE and YE **(C)**, RE **(D)**, and PDE **(E)**. In **(C)** the firing rate is color coded relative to overall mean firing and in relation to the target (white circle). Plots of the simple spike firing with each error parameter based on the optimal τ obtained from the lagged regressions. **(A)** is reproduced from Hewitt et al. (2011) and **(B–E)** from Popa et al. (2012) with permission.

A random tracking task, briefly described in Figures 1A,B, eliminates or reduces these experimental problems and allows for a more systematic evaluation of how arm kinematics are encoded (Paninski et al., 2004). Therefore, we evaluated Purkinje cell firing during random tracking and verified that position, velocity and speed of the limb (denoted in Figure 1B by the C subscripted variables) were statistically independent (Hewitt et al., 2011). For a large majority of Purkinje cells, simple spike firing is modulated in relation to these kinematic parameters. The firing of individual Purkinje cells was characterized using lagged linear regressions of the simple spike firing with kinematics, identifying significant correlations and the timing of the strongest correlations (lead and/or lag). A similar approach has been used by a number of cerebellar investigators (Shidara et al., 1993; Gomi et al., 1998; Medina and Lisberger, 2009). Comparing the regression results based on individual parameters with those based on multiple parameters shows that signals encoding single parameters are mutually independent. Velocity is the dominant parameter, followed by position and then speed (Hewitt et al., 2011; Popa et al., 2012). The lead/lag values, the time intervals by which the neural activity leads (negative values) or lags (positive values) motor behavior, show a negative bias in which the simple spike firing tends to lead motor behavior, as observed in other studies (Marple-Horvat and Stein, 1987; Coltz et al., 1999; Roitman et al., 2005; Medina and Lisberger, 2009). Given the unpredictability of random tracking, this observation strongly supports the assumption that a majority of Purkinje cells predict the kinematic consequences of motor commands. However, the wide distribution of leads and lags, including both negative and positive values, also suggests the presence of both predictive and feedback signals (Marple-Horvat and Stein, 1987; Fu et al., 1997; Roitman et al., 2005). Remarkably, using the coefficients determined during random tracking allowed an accurate reconstruction of the simple spike activity recorded during different tasks, including circular tracking and center-out reach (Hewitt et al., 2011), strongly suggesting that the representations of arm kinematics are task-independent. This implies that a single global limb model is used widely as opposed to requiring large numbers of internal models for specific movements (Wolpert and Kawato, 1998; Imamizu et al., 2003, 2007).

It is possible that the temporal relationships between cerebellar signals and motor behavior reflect hard-wired circuitry properties like synaptic delays and conduction times in which the distribution of lead/lag populations would be normal, centered on well-defined time values. However, the distribution of the lead/lag values of the cerebellar signals during random tracking is extremely broad and quasi-uniform (Hewitt et al., 2011). This observation suggests that the temporal properties of the cerebellar signals are the results of computational processes reflecting the temporal constrains and requirements necessary to execute motor behaviors. For example, as internal model predictions are expected in various motor sequences involved in implementation of motor behavior, the predictions need to be “broadcasted” over a wide range of time intervals. The temporal properties of the kinematic representation are specific to different structures and regions. For example, in the primary motor cortex the distribution of the kinematic leads/lags during a continuous spiral tracing experiment is uni-modal and centered around a 100 ms lead, as determined from signals conveying very accurate representations of the trajectory, while premotor cortex leads/lags are distributed bi-modally around 250 and 0 ms (Moran and Schwartz, 1999). Also, the timing of the signals in the motor cortices are dependent on trajectory curvature (Moran and Schwartz, 1999), while the cerebellar representations are independent of the curvature (Hewitt et al., 2011). These findings highlight fundamental functional differences in kinematic signaling in the cerebellum vs. the motor cortical areas.

### Error processing in the cerebellum—complex spikes or simple spikes?

Error processing has been a long-standing hypothesis of cerebellar function, (Oscarsson, 1980) and there is a long history of studies focused on identifying error-related signals in cerebellar activity. The dominant hypothesis is that the error signals are encoded by the complex spike discharge of Purkinje cells (Oscarsson, 1980; Ito, 2000). An error encoding role of complex spikes has been proposed, not only in the motor domain, but also in the cerebellum's role in non-motor behaviors (Ito, 2008; Schmahmann, 2010; Koziol et al., 2012; Yamazaki and Nagao, 2012). Observations favoring this hypothesis in the motor domain are the complex spike modulation occurring with retinal-slip (Graf et al., 1988; Barmack and Shojaku, 1995; Kobayashi et al., 1998) and induced saccadic errors during eye movements (Soetedjo et al., 2008). Also, complex spike discharge modulates with reach end point errors (Kitazawa et al., 1998), learning a predictable target redirection during smooth pursuit (Medina and Lisberger, 2008), redirection of reaching (Kim et al., 1987), responding to unexpected loads (Gilbert and Thach, 1977), and adaptation to visuomotor transformations (Ojakangas and Ebner, 1994). However, many other experiments found no clear relationship between motor errors and complex spike discharge. Complex spike modulation could not be related to direction or speed errors during center-out reaching (Ebner et al., 2002) nor with eye movement errors during saccade and smooth pursuit learning (Catz et al., 2005; Dash et al., 2010). Also, perturbations and performance errors during reaching in cats failed to evoke responses in inferior olive neurons, the origin of the climbing fiber projection (Horn et al., 1996). An intriguing observation is that complex spike modulation in the oculomotor vermis occurs late in eye movement adaptation and persists after learning has stabilized (Catz et al., 2005; Dash et al., 2010; Prsa and Thier, 2011), which is inconsistent with the traditional error signal hypothesis. Additionally, complex spike error signals occur in a small fraction of trials and are evident only after extensive averaging (Ojakangas and Ebner, 1994; Kitazawa et al., 1998). Also, the very low complex spike firing frequency provides a limited bandwidth to encode the continuous error signals that occur during movements (Ebner et al., 2011).

An open question is whether simple spike discharge encodes errors. Until recently there was limited information on the presence of error signals in the simple spike discharge. In a reaching task, the simple spike activity was modulated with trial success or failure (Greger and Norris, 2005). During manual tracking, simple spike discharge was correlated with direction and speed errors (Roitman et al., 2009), however, the interpretation was confounded because the error parameters were not statistically independent from the kinematics. Instructive signals in the simple spike firing contribute to cerebellar-dependent learning in the vestibulo-ocular reflex (Ke et al., 2009), also suggesting the presence of error signals.

The random tracking paradigm has numerous advantages for addressing these questions about simple spike error encoding. Random tracking involves a high degree of difficulty and the monkeys make frequent excursions outside the target area that require correction in 500 ms to avoid a trial abort (Hewitt et al., 2011). As a result, the task requires continuous evaluation of motor performance and implementing corrective movements to compensate for errors. Performance errors, defined as the divergence between the current movement goal and the consequence of the motor commands, were characterized based on the relative movement between the target center and the hand-controlled cursor. The performance errors evaluated included the cursor position relative to the target center (XE and YE), the distance between cursor and target center (RE) and angular distance from the direction necessary to move from the current position to the target center (PDE) (Popa et al., 2012). These defined performance errors, depicted in Figure 1B, assume that the target center is the current movement goal. Accordingly, the probability density functions of the kinematics and performance measures show the animals strive to keep the cursor in the center of the target and prefer to move toward the target center. It is interesting to note that the error parameters, although related to arm movement, are independent of the kinematic variables, thus forming a novel, non-kinematic class of motor variables related to task execution.

We found that simple spike firing robustly modulates with all four error parameters. As shown for an example Purkinje cell in Figure 1, the simple spike activity increases with both XE and YE, resulting in a planar pattern characterized by increased firing in the upper right quadrant and decreased firing in the lower left quadrant of the circular target (Figure 1C). Firing increases linearly with RE (Figure 1D) and modulates with PDE (Figure 1E), demonstrating that individual Purkinje cells can simultaneously encode a complex representation of performance errors. The frequency of significant error-related modulation was extremely high, with over 90% of Purkinje cells modulated with respect to XE, YE, and RE and over 80% with PDE. In contrast with the view that error coding is relegated to the complex spike discharge, simple spike firing carries a wealth of information about performance errors.

### Dual temporal encoding of errors

**Figure 2:**
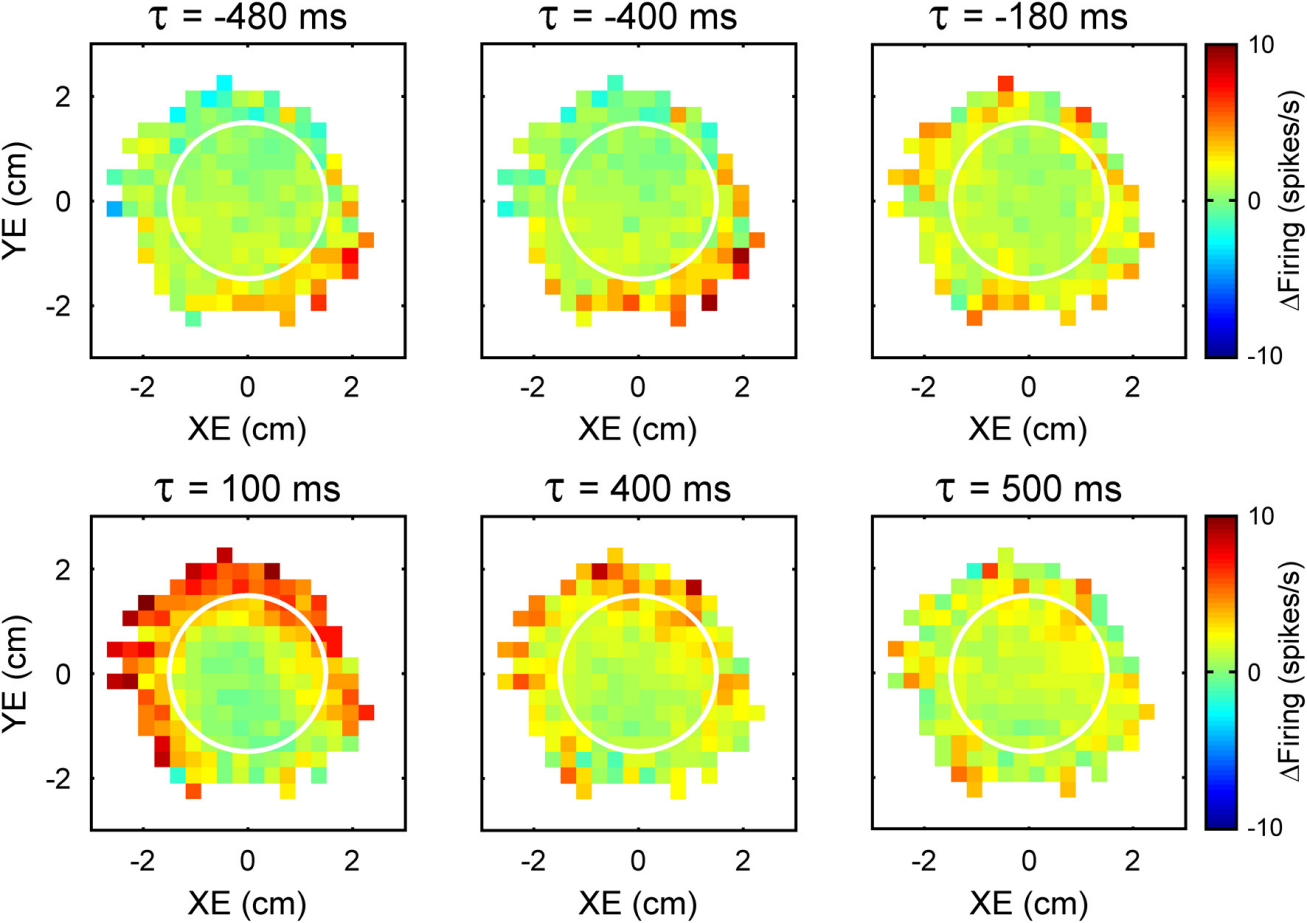
**Time course of the simple spike modulation with performance errors**. Each plot is the simple spike firing in relation to XE and YE as a function of time (τ) from an example Purkinje cell. Negative τ represents the firing leading the error signals. As in Figure 1, simple spike firing rate is color coded relative to overall mean firing and in relation to the target (white circle).

An intriguing aspect of the simple spike modulation with these error parameters is the temporal properties. A natural assumption would be that Purkinje cell firing either leads or lags the behavior by a single, constant time interval at which the firing pattern best correlates with the behavioral parameter. However, examination of the simple spike modulation at different leads and lags reveals a different story. In the example shown in Figure 2, maps of the simple spike firing relative to XE and YE show a modulation pattern characterized by high firing in the lower right quadrant at −480 to −400 ms (i.e., leading the position error) that fades as the time shift approaches 0 ms. Therefore, firing precedes the position errors by 400 ms and suggests simple spike encoding predicts the sensory consequences of the motor command. However, a new modulation pattern emerges at a lag of approximately 100 ms, with high firing at the target edge, except for the lower right quadrant of the error space. This new modulation pattern then fades as the lags approach 500 ms. Therefore, the simple spike firing also lags position error by 100 ms suggesting that firing is modulated by sensory feedback. Interestingly, the modulation patterns at different leads and lags are complementary: the region of high firing at lead time (−400 ms) coincides with the region of low firing at lag time (100 ms). This example shows that encoding of error parameters by individual cells can include both a prediction of the sensory consequences of motor commands as well as sensory feedback.

**Figure 3:**
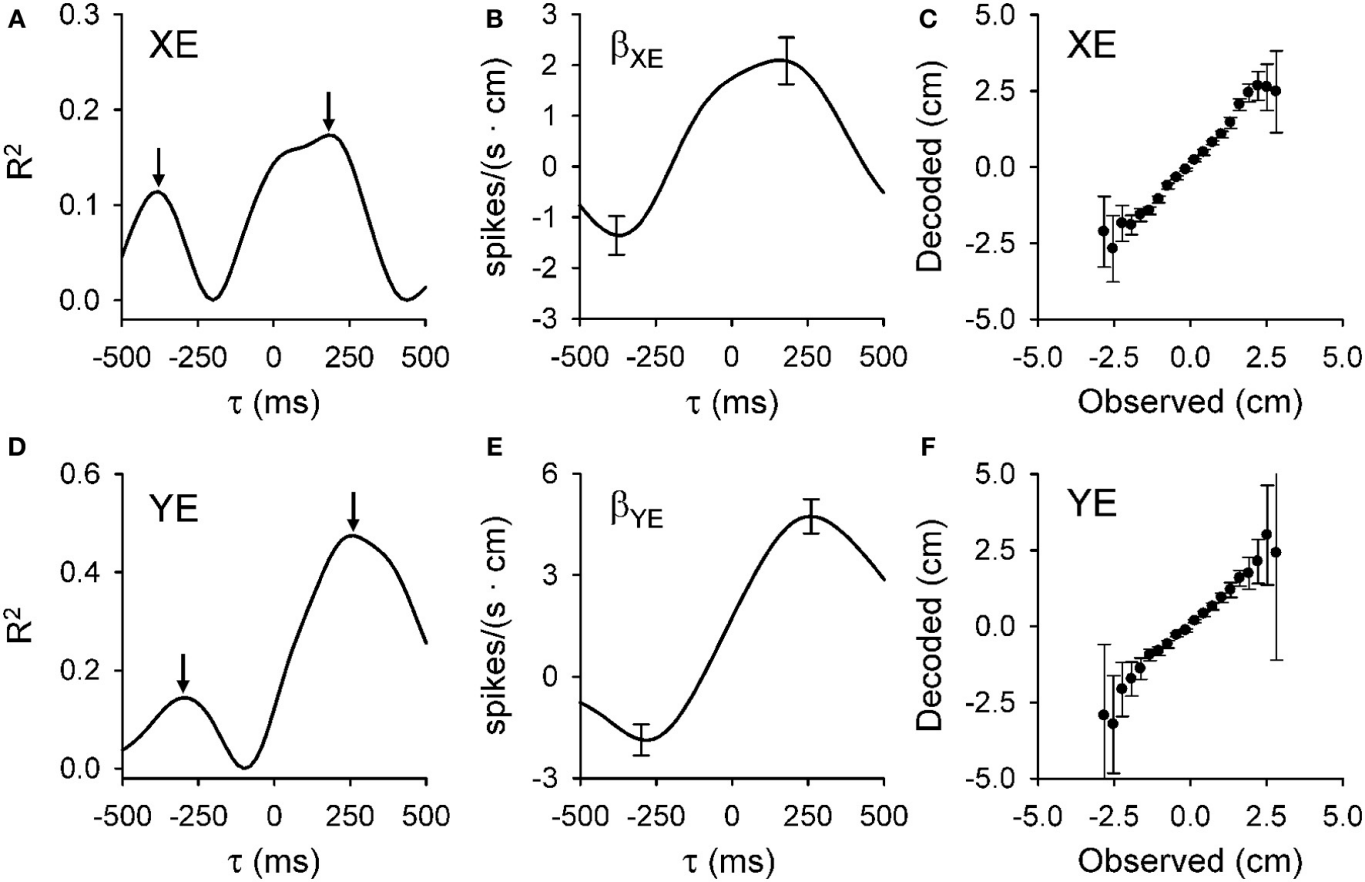
**Dual temporal encoding of error signals. (A,D)** R^2^ as a function of lead/lag (τ) for position error parameters (XE and YE, respectively) from the Purkinje cell shown in Figure 1. **(B,E)** Regression coefficients for XE (β_XE_) and YE (β_YE_) as a function of τ. Arrows denote the times of the significant R^2^ peaks and error bars the confidence intervals of β_XE_ and β_YE_ at the times of the R^2^ maxima [arrows in **(A,D)**, respectively]. Note change in the sign of regression coefficient showing the reversal in the firing sensitivity at predictive versus feedback timing. **(C,F)** Plot of the decoded upcoming XE and YE (mean ± *SD*) versus observed based on the population of Purkinje cells. Slope of decoded estimate versus the observed is 0.99 (ρ = 0.95, *p* < 0.001) for XE and 0.98 (ρ = 0.83, *p* < 0.001) for YE showing the accuracy of the prediction. Decoding estimates were from 25 repetitions of the off-line decoding algorithm (see Popa et al., 2012). Plots are reproduced from Popa et al. (2012) with permission.

The analyses designed to quantify and characterize the encoding of the error variables across the population of Purkinje cells had to clear two hurdles. The first was to capture the complex temporal properties described above. To accomplish this, we used repeated linear regressions of the instantaneous firing against the error parameters at all lags to characterize the temporal relationships between Purkinje cell firing and behavioral variables. The second hurdle was to ensure that the encoding is not due to interactions with other variables, such as kinematics. Here we used linear regressions for each error parameter based on residual firing that was obtained by eliminating the firing variability associated with known variables such as kinematics and the other error parameters (Popa et al., 2012). These analyses yield, as functions of the lead or lag (τ), measures of the goodness of fit [the coefficient of determination (R^2^)] and firing sensitivity [the regression coefficients (β)] for each parameter. Figure 3 exemplifies the R^2^ and β profiles computed only for XE (A,B, respectively) and YE (D,E, respectively) although the cell (the same as in Figure 1) significantly encodes all errors (XE, YE, RE, and PDE). Both R^2^ profiles are bi-modal (Figures 3A,D), with two local maxima. Each R^2^ profile has a peak at negative time values (XE at −400 ms and YE at −300 ms) and a peak at positive time values (XE at 200 ms and YE at 250 ms). Our interpretation of these observations is that XE and YE are dually encoded by predictive and feedback-related signals in the simple spike discharge of a single cell. Importantly, the discharge sensitivity changes sign for both β profiles, with negative values for the predictive representations and positive values for the feedback representations (Figures 3B,E), showing opposing modulations for the predictive and feedback signals encoding the same behavioral parameter. Dual temporal encoding is common. It was observed in 72% of Purkinje cells and 74% of dually encoded parameters show opposing modulations between predictive and feedback signals (Popa et al., 2012). Interestingly, a similar dual encoding mechanism was discovered when monkeys perform a rule processing task where the signals between prefrontal and parietal cortices exhibit dual timing at 50 and 150 ms with anti-correlated modulation (Crowe et al., 2013).

The presence and ubiquity of the dual error signal representations can be interpreted in the context of internal models. Many researchers have postulated that the cerebellum acts as a forward internal model that predicts the sensory consequences of a motor command (Robinson, 1975; Miall and Wolpert, 1996; Wolpert and Ghahramani, 2000; Bastian, 2006; Shadmehr et al., 2010). Integral to implementing a forward internal model is comparing the prediction of the sensory consequences with the actual sensory feedback to compute sensory prediction errors. Sensory prediction errors are the critical signals that drive adaptation of both eye and limb movements (Wallman and Fuchs, 1998; Noto and Robinson, 2001; Mazzoni and Krakauer, 2006; Shadmehr et al., 2010). Functional imaging and patient studies suggest the cerebellum is involved in error processing, including sensory prediction errors (Diedrichsen et al., 2005; Morton and Bastian, 2006; Tseng et al., 2007; Xu-Wilson et al., 2009; Izawa et al., 2012).

Decoding analysis allows one to test whether the simple spike error signals in a population of Purkinje cells contain sufficient information to understand the behavior. Figure 3C,F show the results for decoding the predictions for XE and YE, respectively. Decoded values have highly linear correlations with the observed values for all error variables. Moreover, for XE, YE, and PDE the slope is very close to unity, showing that the Purkinje cell population provides a remarkably accurate prediction of the upcoming errors. As we have noted (Popa et al., 2012), there is some degradation of the decoding accuracy toward the boundaries of the error space that reflects either the relative scarcity of data points or that the predictions are confined to the space relevant for successfully performing the task.

We hypothesized that the dual encoding of performance errors provides the neural substrate needed to generate sensory prediction errors. For the majority of Purkinje cells, the prediction and feedback error signals have opposing effects on simple spike modulations, precisely as required to compute the difference between the predicted and the actual feedback. Dual encoding could result in reduced simple spike sensitivity to self-generated sensory information whereby the presence of both the predicted consequences of the motor commands and sensory feedback signals act to cancel each other. Consistent with this interpretation, Purkinje cells show greater sensitivity to passive self-motion, driven by sensory feedback than to active, self-generated motion driven by both sensory and internal feedback (Brooks and Cullen, 2013). Also, the long term decrease in sensitivity to motor errors observed with PET imaging during adaptation to constant force fields (Nezafat et al., 2001) could be due to an improving match between internally generated predictions and sensory feedback. A similar reduction in the cerebellar BOLD response occurs during a cognitive task in which subjects learn first-order rules (Balsters and Ramnani, 2011), presumably due to a comparison in the cerebellar cortex between the predicted and perceptual consequences of mental manipulations. A study of fear conditioning found that cerebellar activation decreased during the conditioning phase while either unexpected application or omission of the noxious stimuli resulted in increased cerebellar activation (Ploghaus et al., 2000). This result is consistent with acquiring a dual representation of the stimulus application, resulting in cancellation of predictive and feedback signals when the prediction is correct and increased activity when there is prediction error. Furthermore, cerebellar activation during a letter manipulation task is consistent with encoding task execution errors in a non-motor task (Marvel and Desmond, 2012). Although the hypothesis that individual Purkinje cells compute directly sensory prediction errors by subtracting the predictive and feedback signals is very seductive, the fact that the two signals are separated in time requires further investigation. It is possible that this computation is performed downstream, for example, in the cerebellar nuclei. It is also possible that for relatively slow changing signals the dual encoding could approximate the sensory prediction error.

Similar cancellation of self-generated sensory signals occurs in cerebellum-like structures (Bell et al., 2008; Sawtell and Bell, 2008; Requarth and Sawtell, 2011). This suppression is due to anti-Hebbian plasticity at the parallel fiber-principle neuron synapse that sculpts the response to the efferent copy into a negative image of the response to the sensory input (Bell et al., 1997; Han et al., 2000; Requarth and Sawtell, 2011). This mechanism is predicated on the appropriate convergence of sensory inputs and an efference copy of the motor command on the principal neurons (Sawtell, 2010). A comparable convergence of proprioceptive and cortico-pontine inputs has been reported in the mouse cerebellar cortex (Huang et al., 2013).

To test whether dual encoding is a general principle of cerebellar representations, we re-examined the simple spike firing relationship with kinematics using the analytical methods described above, regressing the appropriate residuals in which variability associated with all other motor parameters was removed against individual kinematic variables (Popa, 2013). Similar to the error parameters described earlier, a majority of Purkinje cells also encode kinematics with dual signals, one related to an internal prediction and one related to sensory feedback. There is increasing evidence that the time delays required to create opposing signals might be generated by the mossy fiber-granule cell circuitry in the mammalian cerebellum. Long term plasticity at the mossy fiber—granule cell synapse can delay incoming signals for up to 100 ms, or even longer when larger sections of the circuitry are entrained (D'Angelo, 2011). Similar delays are generated in cerebellar-like structures involving the mossy fibers, granule cells and unipolar brush cells (Kennedy et al., 2014). Together, these findings suggest that the leads/lags found for performance errors and kinematic signals are determined, at least in part, by computations occurring in the cerebellar cortex, resulting in specific temporal alignments of cerebellar signals. We hypothesize that dual temporal encoding is a general property of cerebellar cortical information processing and also likely plays a central role in non-motor cerebellar functions.

Both motor coordination and motor sequences require interactions between different components (e.g., effectors, muscles, etc.) activated at different times and, therefore, controlling the individual components requires motor predictions across a spectrum of leads. Successful population decoding of the error predictions combines individual Purkinje cell signals at all possible lead times, with values between -500 and 0 ms. In other words, the cerebellar cortex unfurls the predictions throughout a long time window. Similarly, the simple spike signals that lag kinematics or errors occur across this rather protracted time course. One possible interpretation is that this provides a mechanism for the cerebellum's role in coordination among effectors (Thach et al., 1992; van Donkelaar and Lee, 1994; Bastian et al., 1996; Serrien and Wiesendanger, 2000; Miall et al., 2001) and in movement sequences (Braitenberg et al., 1997; Doyon et al., 1997; Molinari and Petrosini, 1997; Molinari et al., 1997, 2008; Nixon and Passingham, 2000). Under this assumption, cerebellar cortical output integrates the predicted motor command outcomes at specific lead times with appropriately matched feedback signals to generate the required coordination among effectors needed to accomplish the movement goal while simultaneously updating the motor controller so that the next motor command can be generated. It has been hypothesized that the cerebellum fulfills a similar function in detecting sequences in the verbal, spatial and cognitive domains (Molinari et al., 2008). The long and quasi-uniform lead and lag times found in the simple spike discharge provides a neural substrate for monitoring and controlling sequences. For example, cerebellar damage impairs sequencing of cards depicting brief stories regardless of whether verbal, spatial, or behavioral strategies are used (Leggio et al., 2008). An imaging study based on letter manipulations showed an interesting difference in cerebellar activation depending on the cognitive process engaged (Marvel and Desmond, 2012). The presentation of a sequence of letters, without processing requirements, induces a fast, transient activation, consistent with the response to tightly packed input signals. However, when processing instructions are added, the activation becomes sustained over a longer time interval. This temporal expansion is consistent with the temporal unfurling of the cerebellar representations observed during random tracking.

Another interesting implication for the capacity of Purkinje cells to provide predictions and feedback at a wide range of times is in working memory. Numerous functional imaging studies demonstrate cerebellar activation associated with the working memory system (Chen and Desmond, 2005; Hautzel et al., 2009; Marvel and Desmond, 2010). Given that the storage capacity of working memory is limited to between four and seven items (Miller, 1956; Luck and Vogel, 1997), the changes in its content associated with shifting attention focus occurs every 200–500 ms (Muller et al., 1998; Woodman and Luck, 1999). Having both prediction and feedback signals over comparable time horizons, the cerebellar cortex may facilitate novel associations between past and current working memory content and among different classes of information.

### Linear integration of kinematics and error signals

The presence of both error and kinematic signals in the Purkinje cell simple spike discharge raises the question of whether the cells are functionally segregated into populations that preferentially encode one class of signals over the other. Using multi-linear models that included (1) all variables from each class, (2) position, velocity, and speed kinematics, and (3) position, radial, and direction errors, we showed that the average R^2^ for kinematics and error models are comparable. As detailed above, accurate decoding of the upcoming behavior can be achieved for both errors and kinematics. Therefore, encoding of these two classes of variables is equally robust. For single cells, the distribution of R^2^ values reveals a strong linear positive correlation between errors and kinematics and there is no evidence of segregation into subpopulations. The representations of single parameters are additive, as the sum of the R^2^ profiles for the individual parameters closely matches the R^2^ profile of both error and kinematic multi-linear models (Popa et al., 2012; Popa, 2013). These observations reinforce the concept of signal independence and supports the hypothesis that Purkinje cells linearly integrate parallel fiber input (Walter and Khodakhah, 2006, 2009). Therefore, kinematic and error signals are highly integrated at the single neuron and population levels.

The integration of the kinematic and error signals strongly suggests that Purkinje cells favor complexity. There are approximately 200,000 parallel fiber-Purkinje cell synapses (Napper and Harvey, 1988), while less than 200 active synapses are required to drive simple spike discharge (Isope and Barbour, 2002), suggesting a very high theoretical bandwidth. During random tracking, we evaluated nine behavioral parameters, five kinematic and four error signals. Each parameter could be dually encoded, resulting in 18 independent signals in the simple spike discharge. Out of these 18 possible signals, on average, individual Purkinje cells encode 10 different signals simultaneously (Popa, 2013). Therefore, each Purkinje cell carries a very rich representation of the motor behavior suggesting that an important aspect of cerebellar function is associating different signals, possibly of different modalities.

The number of signals identified in individual cell simple spike discharges is small in comparison to the theoretical bandwidth, suggesting the possibility that the same cell could encode diverse representations in different contexts. For example, one cell could use a common representation of an effector with multiple performance error signals that differ across tasks. As reviewed above, functional imaging studies show that the cerebellum encodes errors as well as kinematics. Although clearly not at the level of single cells, imaging suggests the cerebellum is activated in relation to non-motor functions including language, spatial processing, working memory, executive function, and emotional processing (for review see Stoodley, 2012). These activations particularly engaged the postero-lateral regions and are consistent with the integration of many classes of signals within the same areas. Association of information across modalities has been central to several theories of the cerebellum's role in cognition (Drepper et al., 1999; Timmann et al., 2002, 2010; Molinari et al., 2008).

The high number of independent signals present in each Purkinje cell discharge and the additive property of these signals suggest that the cerebellar function includes an associative aspect. As others have proposed, it is possible that Purkinje cells use plasticity mechanisms to select only the relevant signals from the high number of possible parallel fiber inputs to each neuron, and these relevant signals maintain consistent relationships to form a representation of the motor behavior (Marr, 1969; Albus, 1971). Associative learning in the cerebellum is not restricted to the motor domain (for review see Timmann et al., 2010). For example, the cerebellum is implicated in fear learning (Sacchetti et al., 2004) and cognitive associative learning (Drepper et al., 1999; Timmann et al., 2002). Making the required association between the relevant afferent signals would utilize the Purkinje cell's capacity to integrate large numbers of signals. Interestingly, the cerebellum appears to acquire an internal model related to the application of noxious stimuli but fails to respond when innocuous stimuli are applied (Ploghaus et al., 2000), suggesting that the cerebellum evaluates the relevance of inputs and disregards the irrelevant ones.

## Implications for internal models of cognitive processes

Understanding cerebellar involvement in non-motor domains faces numerous challenges ranging from imprecise definitions and imperfect models of cognitive behavior to indirect investigation methods (Koziol et al., 2012; Buckner, 2013). Fortunately, the remarkable uniformity of the cerebellar cortex architecture (Eccles et al., 1967; Ito, 1984; Ramnani, 2006) suggests that the cerebellum should perform the same signal processing across motor and non-motor domains (Ramnani, 2006; Thach, 2007; Ito, 2008; Schmahmann, 2010). Under this hypothesis, insights into cerebellar processes provided by single cell studies of motor behavior in well controlled experimental conditions and within a rigorous theoretical framework may help illuminate non-motor aspects of cerebellar function.

Throughout this review we have noted how the information processing properties of cerebellar neurons might contribute to non-motor functions. In this final section we explore more fully the concept of internal models in motor and non-motor functions of the cerebellum. Several investigators have proposed that the internal model hypotheses can be used to understand cerebellar involvement in non-motor domains (Ito, 2008; Imamizu and Kawato, 2009; Koziol et al., 2012). In support of the hypothesis that forward internal model processes are common across function domains is the observation that the information provided by an internal model of the hand during visually guided tracking is used in a visual discrimination task (Stanley and Miall, 2009) showing that signals involved in motor control are used in non-motor behaviors. It also been hypothesized that forward internal models acquired in the motor domain, interacting with the mirror system in the cerebral cortex, facilitate action understanding (Caligiore et al., 2013), the capability to asses mental states such as goals and intentions underlying actions performed by different subjects. Several functional imaging studies found specific cerebellar activation in response to noun-verb associations (Petersen et al., 1988) or to word completion tasks (Desmond and Fiez, 1998), suggesting activation of a forward internal model that provides lexical predictions. Strongly supporting this hypothesis is the observation that disruption of cerebellar function by repeated transcranial magnetic stimulation reduces language predictive performance (Lesage et al., 2012). Under the assumption that cerebellar processing in the motor domain translates to non-motor functions, we consider the implications of our random tracking results to understanding the cerebellar contribution to cognitive processes in the context of forward internal models. The classical version of the forward internal model assumes that the cerebellum provides a model of the effector that predicts the sensory consequences of the motor commands (Robinson, 1975; Miall and Wolpert, 1996; Bastian, 2006; Shadmehr et al., 2010). In this framework, the internal model updating is driven by the motor error signals, more specifically sensory prediction errors (Morton and Bastian, 2006; Tseng et al., 2007; Xu-Wilson et al., 2009; Shadmehr et al., 2010; Izawa et al., 2012). As detailed above, the historical emphasis has been that the error signals are conveyed solely by the climbing fibers and complex spikes. Likewise, it has been assumed that complex spikes provide error signals in non-motor behaviors (Ito, 2008; Schmahmann, 2010; Koziol et al., 2012; Yamazaki and Nagao, 2012). It has also been proposed that the cerebellum uses the same processes performed by a forward internal model on copies of the mental models encoded in the parietal cortex which are then manipulated by the commands issued by the prefrontal cortex, while the inferior olive encodes the cognitive errors (Ito, 2008). Recently described projections between the cerebral cortex and cerebellum provide the required pathways to support the hypothesis that the cerebellar cortex acquires and manipulates copies of the mental models (Schmahmann and Pandya, 1989, 1991, 1997; Middleton and Strick, 1994, 2001; Kelly and Strick, 2003). However, the existence of pathways necessary to support the hypothesis that the inferior olive encodes cognitive error signals remain tentative at best (Ito, 2008). For example, principal olive, the subdivision of the inferior olive that provides the climbing fiber projections to cerebellar cortical regions connected to associative areas of the cerebral cortex, does not receive projections from the cerebral cortex (Schmahmann, 2010). Further, as discussed above, the validity of the concept that complex spikes signal motor errors is being questioned.

Our recent findings describing performance error signals in the simple spike activity show that the signals required by the forward internal model theories are conveyed by parallel fiber inputs, thus eliminating the idea that climbing fibers are the sole pathway for conveying error signals (Ito, 2008). This would strengthen the hypothesis that forward internal model processes could be replicated in the cognitive domain by emphasizing the established projections between cerebellum and prefrontal and parietal cortices and reducing the relevance on inferior olive input. However, this would also require a reassessment of the role of complex spikes in the cerebellar function. For example, recent optogenetic experiments show that simple spike activation induces a complex spike response (Chaumont et al., 2013; Witter et al., 2013). This suggests that the complex spike activity, modulated by the response of Purkinje cells to parallel fiber inputs, engage specific plasticity mechanisms (Ito et al., 1982; Marquez-Ruiz and Cheron, 2012) that refine suboptimal outputs of the cerebellar cortex.

Complex goal-directed behavior, such as visually guided, random tracking, raises the question of how a motor command is selected and implemented under very stringent temporal constraints as the monkey has only 500 ms to recover from excursions outside the target. Within such a tight temporal budget it is unlikely that a classical process of error perception and action selection, which requires ~400 ms, can be implemented (Madl et al., 2011). One accepted solution to this problem is that the cerebellum acts as a forward internal model that predicts the future state of the effector (Ito, 2008; Shadmehr and Krakauer, 2008). Nevertheless, the selection of the motor command cannot be completed based only on effector state predictions, as the action selection is also dependent on performance information. This information is presumably processed in the classical sequence of perception-selection-execution. However, our demonstration of the integration of kinematic and performance error predictions shows that the cerebellar cortex provides predictions of the consequences of the motor command both in terms of future states of the effector and the usefulness toward meeting the current movement goal. We suggest that the cerebellar cortex predictions provide the information needed to complete action selection with little reliance on sensory feedback.

First, it is interesting to explore the implications of cerebellar prediction of both kinematic and performance states for the process of action selection that involves interactions among widely distributed cortical areas and also subcortical structures including the cerebellum and basal ganglia (Cisek and Kalaska, 2010). Action selection appears to be heavily dependent on perception (Ledberg et al., 2007) and therefore, may be too slow for the implementation and control of fast, ongoing behaviors, such as random tracking. Decision making may be adequate to control the slower components of the behavior, such as the decision to start a new trial or to abandon an ongoing trial. However, once the decision to engage in a trial is made, the selection and implementation of the on-going movements required to complete the behavior needs to be controlled by faster mechanisms (Shadmehr et al., 2010). Faster control could be achieved by acquiring a forward internal model of the behavior that combines signals describing the effector response and the task-specific performance and then learning to predict the consequences of the specified actions. Therefore, action bias might be viewed as a staged process in which a slower, perception-driven component are under prefrontal and basal ganglia control, while the cerebellum controls a faster, experience-dependent and perception-independent component.

Second, it is interesting to examine the cerebellar internal model of thought process hypothesis (Ito, 2008) in an expanded version of the forward internal model framework. It had been suggested that when the cerebellum acquires a forward internal model of a cognitive problem, by performing the manipulations required to solve the problem on cerebellar copies of the mental models eliminates the perception of thinking about the problem, thus generating an “intuition” (Ito, 2008). However, this model leaves unanswered the problem of recognizing the solution. It is possible to perform the evaluation of the intermediate results under conscious control, which would eliminate the “unexpected” quality associated with intuition. An internal model that includes not only copies of the mental models manipulated in the process, but also copies of cognitive error representations, could allow the evaluation of the results under cerebellar control.

Extending the internal forward model hypothesis to both predictions about the effectors and performance could have another implication. Presumably, experience-driven internal models are acquired by consistently activating and associating specific cerebellar inputs and the required plasticity mechanisms. Under normal conditions the consistency of inputs would be only related to repeated consideration of the same problem and, therefore, the resulting internal models could be refined to provide an optimal solution. However, if unrelated inputs are consistently activated, for example in pathological conditions, this could bias the internal forward model. In this condition, the subject would perceive a suboptimal result as the correct solution.

## Next steps

Extending cerebellar function into non-motor processing has raised a host of intriguing questions about the computations performed by the cerebellar circuitry and how these computations support processes such as cognition, working memory, and language processing. While patient studies, non-invasive stimulation techniques and functional imaging in human subjects are powerful tools to test hypotheses on the role of the cerebellum in non-motor functions and have already provided considerable insights, single unit electrophysiological studies are clearly needed to gain a greater understanding of how the cerebellum participates in and contributes to higher cortical functions at the neuronal level. Electrophysiological studies would use non-human primates because these subjects are capable of the types of complex, non-motor behaviors needed to address these questions. Furthermore, non-human primates have the required cerebellar-cortical connectivity that underpins the cerebellum's role in non-motor behaviors. Finally, single cell recordings are the only present day technique that can answer the question central to this review, which is “Are the computations performed by the cerebellar cortex similar in the motor and non-motor domains?”

## Author contributions

Angela L. Hewitt, Laurentiu S. Popa, and Timothy J. Ebner all contributed to the design, analysis, and interpretation of the work. Angela L. Hewitt had primary responsibility for data acquisition. All authors contributed to the drafting, giving final approval and agreeing to be held accountable for all aspects of the work.

### Conflict of interest statement

The authors declare that the research was conducted in the absence of any commercial or financial relationships that could be construed as a potential conflict of interest.
